# Urgent Endoscopic Retrograde Cholangiopancreatography (ERCP) vs. Conventional Approach in Acute Biliary Pancreatitis Without Cholangitis: An Updated Systematic Review and Meta-Analysis

**DOI:** 10.7759/cureus.21342

**Published:** 2022-01-17

**Authors:** Dhan B Shrestha, Pravash Budhathoki, Yub Raj Sedhai, Anurag Adhikari, Ayusha Poudel, Barun B Aryal, Tul Maya Gurung, Binod Karki, Bhesh Raj Karki, Dhruvan Patel

**Affiliations:** 1 Department of Internal Medicine, Mount Sinai Hospital, Chicago, USA; 2 Department of Internal Medicine, BronxCare Health System, Bronx, USA; 3 Department of Internal Medicine, Virginia Commonwealth University School of Medicine, Richmond, USA; 4 Intensive Care Unit, Nepal Korea Friendship Municipality Hospital, Madhyapur Thimi, NPL; 5 Department of Emergency Medicine, Alka Hospital Pvt. Ltd., Kathmandu, NPL; 6 Department of Emergency Medicine, B.P. Smriti Hospital, Kathmandu, NPL; 7 Department of Surgery, Nepal Medical College, Kathmandu, NPL; 8 Department of Gastroenterology, Nepalese Army Institute of Health Sciences, Kathmandu, NPL; 9 Department of Internal Medicine, SUNY (State University of New York) Downstate Health Sciences University, New York, USA; 10 Department of Gastroenterology, Mercy Catholic Medical Center, Darby, USA

**Keywords:** mortality, meta-analysis, pancreatitis, cholangitis, endoscopic retrograde cholangiopancreatography

## Abstract

Gallstone disease is the common cause of acute pancreatitis. The role of early endoscopic retrograde cholangiopancreatography (ERCP) in biliary pancreatitis without cholangitis is not well-established. Thus, this study aims to compare the outcome of early ERCP with conservative management in patients with acute biliary pancreatitis without acute cholangitis. An online search of PubMed, PubMed Central, Embase, Scopus, and Clinicaltrials.gov databases was performed for relevant studies published till December 15, 2020. Statistical analysis was performed using RevMan v 5.4 (The Nordic Cochrane Centre, Cochrane Collaboration, Copenhagen). Odds Ratio (OR) with a 95% confidence interval was used for outcome estimation. Among 2700 studies from the database search, we included four studies in the final analysis. Pooling of data showed no significant reduction in mortality (OR 0.59, 95% CI 0.32 to 1.09; p=0.09); overall complications (OR 0.56, 95% CI 0.30 to 1.01; p=0.05); new-onset organ failure (OR 1.06, 95% CI 0.65 to 1.75; p=0.81); pancreatic necrosis (OR 0.80, 95% CI 0.49 to 1.32; p=0.38); pancreatic pseudo-cyst (OR 0.44, 95% CI 0.16 to 1.24; p=0.12); ICU admission (OR 1.64, 95% CI 0.97 to 2.77; p=0.06); and pneumonia development (OR 0.81, 95% CI 0.40 to 1.65; p=0.56) by urgent ERCP comparing with conventional approach for acute biliary pancreatitis without cholangitis. Henceforth, early ERCP in acute biliary pancreatitis without cholangitis did not reduce mortality, complications, and other adverse outcomes compared to the conservative treatment.

## Introduction and background

Acute pancreatitis (AP) is the most common pancreatic disease worldwide and one of the most common gastrointestinal causes of hospital admission [1,2]. The most common cause of AP is gallstones [3]. Impacted biliary stones and biliary sludge can cause reflux of pancreatic enzymes into the pancreas or cause transient obstruction of the ampulla, leading to inflammation of the pancreas [4]. Possible complications of AP include infection, pseudocyst, cholangitis, organ failure, etc. [5,6].

Conservative management for AP includes fluid replacement, pain control, input/output monitoring, nutritional support via the enteral or parenteral route, and antibiotics in selected cases. Endoscopic retrograde cholangiopancreatography (ERCP) is a therapeutic modality in several hepatobiliary diseases, including patients with biliary AP. Several observational studies and clinical trials have been performed comparing conservative management with ERCP in patients with biliary AP [7-12]. Relatively fewer studies have been conducted focusing only on patients with biliary AP without concomitant cholangitis. A meta-analysis conducted in 2008 found that early ERCP did not cause a significant reduction in the risk of overall complications and mortality in cases of AP without cholangitis [13]. More studies have been published since, with conflicting results [10,11]. The American Gastroenterological Association Institute Technical Review in 2018 recommended ERCP to be performed between 24-48 hours after the diagnosis of acute biliary pancreatitis but did not specify the timing of ERCP in patients with acute pancreatitis without concomitant cholangitis and recommends further studies on this topic [14].

While there is a universal agreement regarding an early ERCP within 24 hours in biliary AP complicated by cholangitis, the utility of an early ERCP in AP without cholangitis remains unclear. This study thus aims to compare the outcome of early ERCP with conservative management in patients with acute biliary pancreatitis without acute cholangitis.

## Review

Objectives

This study aims to determine the usefulness of early ERCP in the management of acute biliary pancreatitis without concomitant cholangitis by comparing the outcomes reported in previous studies such as mortality, local and systemic complications, and hospital stay between patients undergoing early ERCP (within 72 hours) to patients who were managed conservatively.

Methodology

This study was conducted using the Preferred Reporting Items for Systematic Reviews and Meta-Analyses (PRISMA) guidelines [15]. In addition, the study protocol was registered in the international prospective register of systematic reviews (PROSPERO ID: CRD42021226022) [16].

Criteria for considering studies for this review

Types of Studies

In the initial review, we included all case studies (with five or more cases), cross-sectional studies, case-control studies, cohort studies, and clinical trials focusing on patients with acute biliary pancreatitis without concomitant cholangitis. We also included clinical trials in which the sequelae for cholangitis were given separately.

Types of Participants

Patients with acute biliary pancreatitis without cholangitis who were managed with either early ERCP (within 72 hours of presentation) or conservatively (e.g., no ERCP) were included in the study.

Types of Interventions

Patients diagnosed with acute biliary pancreatitis who underwent ERCP within 72 hours of presentation were included in the intervention group. Those who were managed conservatively were included in the control group.

Types of Outcome Measures

Patient characteristics on admission were analyzed, including demographics, clinical status, the severity of pancreatitis, laboratory parameters, including serum bilirubin, serum aminotransferases, and alkaline phosphatase. Mortality, local and systemic complications were also compared.

Outcomes

In-hospital mortality was the primary outcome of the study. Rates of local and systemic complications, including new-onset organ failure, pneumonia, pancreatic necrosis and pseudocyst, and ICU admission, were secondary outcomes of interest.

Search methods for identification of studies

An online search of PubMed, PubMed Central, Embase, Scopus, and Clinicaltrials.gov databases was performed for studies published till December 15, 2020. Two reviewers independently performed searches which were then combined. MeSH headings included “Cholangiopancreatography, Endoscopic Retrograde”, “Pancreatitis”, “Pancreatitis, Acute Necrotizing”, and “Cholangitis”. Next, the title/abstract review followed by the full-text review was performed independently by two reviewers using the Covidence service. A third reviewer resolved conflicts in both steps. Finally, data extraction and review of bias were performed following a full-text review.

Electronic searches

The detailed search strategy has been attached in Appendix 1.

Data collection and analysis

RevMan 5.4 software (The Nordic Cochrane Centre, Cochrane Collaboration, Copenhagen) was used to analyze the data extracted from the selected studies. First, the heterogeneity among the studies was determined using the I^2^ test. Then, a random/fixed-effect model was used based on heterogeneity to pool the various studies appropriately.

Selection of studies

The qualitative analysis included all studies where the patient either underwent early ERCP or was managed conservatively. Quantitative analysis included studies with intervention (early ERCP) and control groups. Case studies with less than five cases, editorials, opinions, letters to the editor, animal studies, studies published in other languages with no English translation were excluded.

Data extraction and management

The quality of the included studies was assessed vigorously.

Assessment of risk of bias in included studies

**Figure 1:**
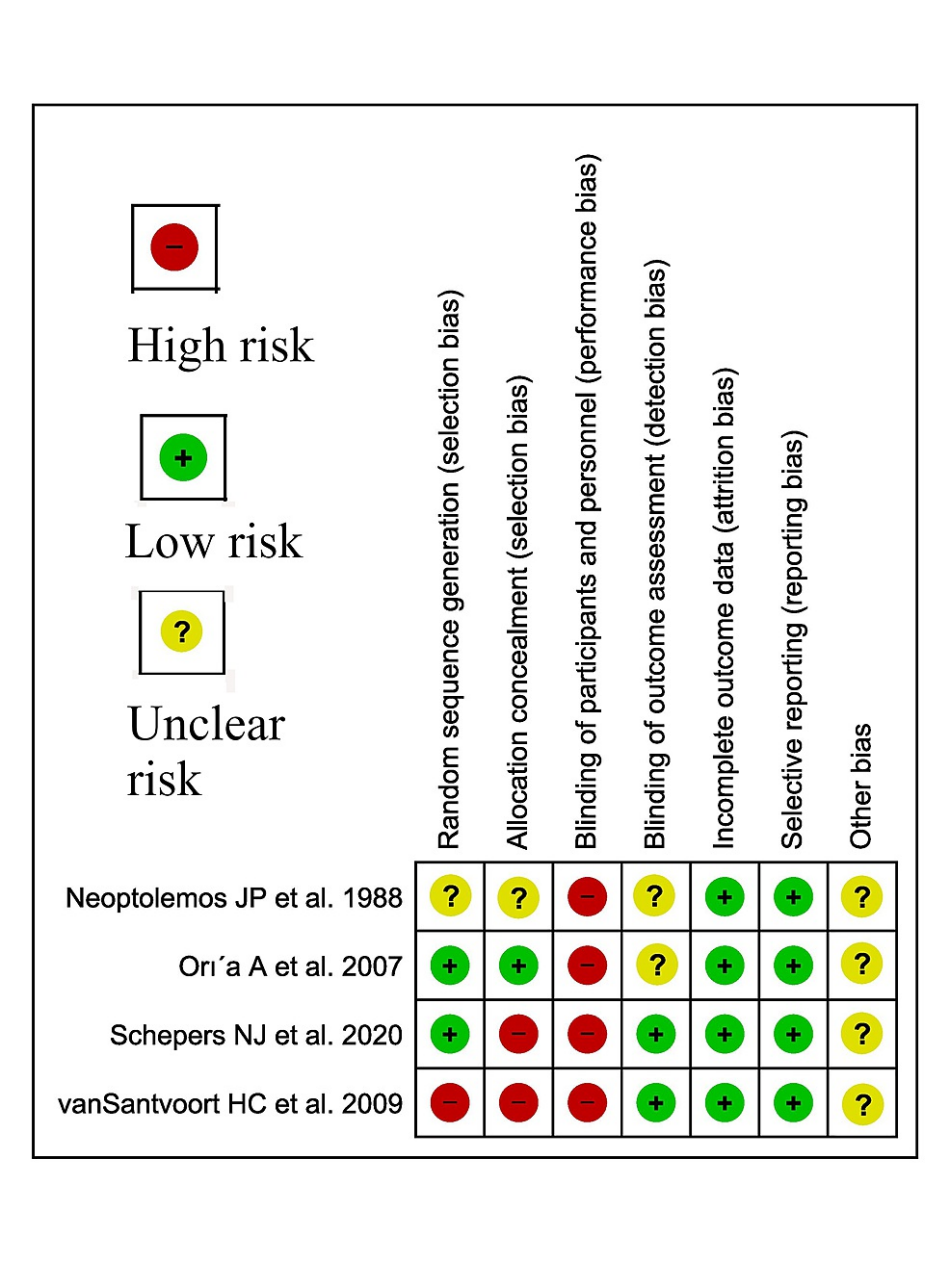
Cochrane Risk of Bias (RoBs) of included studies Four studies were included in the analysis [7,9-11].

Cochrane risk of bias (ROB) was used for the assessment of bias in trials (Figure 1) [17].

Assessment of heterogeneity

The I^2^ test was used to assess heterogeneity using the Cochrane Handbook for Systematic Reviews of Interventions [18].

Assessment of reporting biases

Reporting bias was checked by prefixed reporting of the outcome.

Data synthesis

Statistical analysis was performed using RevMan v 5.4. Odds Ratio (OR) with a 95% confidence interval was used for outcome estimation. In addition, a random/fixed-effects model was used to pool data due as appropriate based on heterogeneity.

Sensitivity analysis

Sensitivity analysis was performed by analyzing the results of randomized controlled trials (RCTs) alone, excluding retrospective studies.

Results

**Figure 2:**
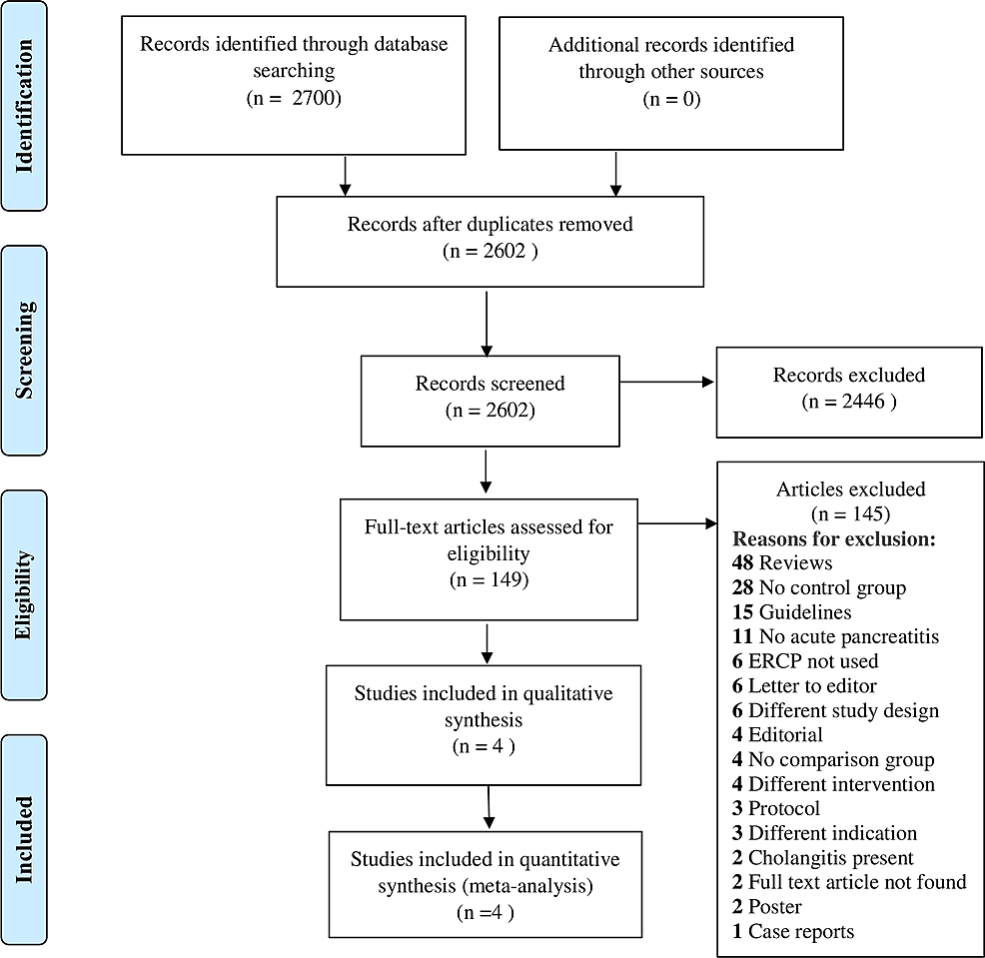
PRISMA Flow diagram n: number; ERCP: endoscopic retrograde cholangiopancreatography

We identified 2700 studies after thorough database searching and removed 98 duplicates. Title and abstracts of 2602 studies were screened. We excluded 2446 studies after the title and abstract review did not meet our inclusion criteria, and assessed the full text of 149 studies. A total of 145 studies were excluded for definite reasons (Figure 2). We included four studies in the final qualitative analysis (Table 1) and quantitative analysis. Basic study details are attached in Appendix 2.

**Table 1:** Qualitative summary RCT: randomized controlled trial; ERCP: endoscopic retrograde cholangiopancreatography; CBD: common bile duct; IV: intravenous; SD: standard deviation; n: number; APACHE: acute physiology and chronic health evaluation; IQR: interquartile range; mg: milligrams; L: liters; ICU: intensive care unit; ES: endoscopic sphincterotomy; DIC: disseminated intravascular coagulation; dL: deciliters *Also includes patients with acute cholangitis, data for only non-cholangitis patients not available

Study ID	Particulars	Intervention group	Comparator group
Schepers NJ et al. [10]	Year	2020	
	Study design	RCT	
	Total participants	230	
	Description	Early ERCP with sphincterotomy within 72 hours after symptom onset and 24 hours of hospital admission irrespective of presence of CBD stones; no antibiotic prophylaxis	IV fluids, analgesics, enteral nutrition, treatment of endocrine and exocrine pancreatic insufficiency, and gastric tube as necessary; no antibiotic prophylaxis
	Population characteristics		
	Participants	117	113
	Male (number/total)	66/117	60/113
	Mean age (± SD) (years)	69±13	71±12
	Cholestasis at admission, n (%)	63 (54%)	67 (59%)
	APACHE-II at admission, median (IQR)	11 (9–15)	10 (8–13)
	C-reactive protein, median (IQR) (mg/L)	60 (13–166)	38 (11–104)
	Outcome		
	Mortality within six months (number/total)	8/117	10/113
	Major complication within six months (number/total)	37/117	40/113
	New-onset organ failure (number/total)	22/117	17/113
	Cholangitis (number/total)	2/117	11/113
	Bacteremia (number/total)	17/117	25/113
	Pneumonia (number/total)	9/117	10/113
	Pancreatic parenchymal necrosis (number/total)	17/117	18/113
	Pancreatic insufficiency (number/total)	9/117	3/113
	Readmission for gallstone-related complication (number/total)	14/117	24/113
	Hospital stay (days, median)	13 (9-24)	14 (10-26)
	ICU admission (number/total)	24/117	13/113
	ICU stay (days, median)	6 (4-17)	8 (4-35)
Neoptolemos JP et al. [7]	Year	1988	
	Study design	RCT	
	Total participants	110	
	Description	Urgent ERCP +/- ES within 72 hours of presentation, a cephalosporin; IV fluids, oxygen, and assisted ventilation as needed	A cephalosporin; IV fluids, oxygen, assisted ventilation as needed
	Population characteristics		
	Participants	53	57
	Male (number/total)	25/59*	27/62*
	Outcome		
	Mortality (number/total)	0/53	5/57
	Overall complications (number/total)	6/53	19/57
	Pseudo-cyst (number/total)	5/53	12/57
	Duodenal obstruction (number/total)	0/53	1/57
	Ascites (number/total)	0/53	1/57
	Portal venous thrombosis (number/total)	0/53	1/57
	Pleural effusion (number/total)	0/53	4/57
	Respiratory failure (number/total)	2/53	7/57
	Cardiovascular failure (number/total)	1/53	5/57
	Renal failure (number/total)	0/53	2/57
	DIC (number/total)	1/53	1/57
	Cerebrovascular accident (number/total)	1/53	1/57
Orı´a A et al. [9]	Year	2007	
	Study design	RCT	
	Total participants	102	
	Description	ERCP +/- ES within 72 hours of onset, ciprofloxacin and metronidazole prophylaxis	ciprofloxacin and metronidazole prophylaxis; IV fluids, analgesia, oxygen, and nasogastric intubation as needed
	Population characteristics		
	Participants	51	51
	Male (number/total)	16/51	13/51
	Mean age (± SD) (years)	49.9 ± 17.4	44 ± 17.7
	Distal bile duct diameter (± SD) (mm)	10.7±2	10.7±2.4
	Total serum bilirubin (± SD) (mg/dL)	3.16±2.1	4±3.3
	APACHE II score (± SD)	4.6±2	4±3.2
	Predicted mild attacks (number/total)	34/51	30/51
	Predicted severe attacks (number/total)	17/51	21/51
	Outcome		
	Mortality within three months (number/total)	3/51	1/51
	Organ failure (newly developed) (number/total)	5/51	6/51
	Pseudo-cyst (number/total)	7/51	9/51
	Renal failure (number/total)	2/51	0/51
	Coagulation failure (number/total)	2/51	1/51
	Cardiovascular failure (number/total)	1/51	0/51
	Infected necrosis (number/total)	2/51	2/51
	Acute pseudocyst (number/total)	1/51	1/51
	Perforated gallbladder/empyema (number/total)	3/51	2/51
vanSantvoort HC et al. [11]	Year	2009	
	Study design	Non-randomized trial	
	Total participants	153	
	Description	ERCP within 72 hours of onset	No ERCP or ERCP later than 72 hours of onset
	Population characteristics		
	Participants	81	72
	Male (number/total)	34/81	38/72
	Mean age (± SD) (years) (patients with cholestasis)	64.1 ± 15.7	66.3 ± 13.3
	Mean age (± SD) (years) (patients without cholestasis)	62.9 ± 15.6	65.9 ± 15.5
	Total serum bilirubin (± SD) (mg/dL) (patients with cholestasis)	4.0 ± 2.7	1.4 ± 0.5
	Total serum bilirubin (± SD) (mg/dL) (patients without cholestasis)	4.6 ± 2.8	1.3 ± 0.5
	Outcome		
	Mortality within three months (number/total)	7/81	12/72
	Overall complications (number/total)	26/81	33/72
	Pancreatic necrosis (number/total)	18/81	21/72
	Infected pancreatic necrosis (number/total)	9/81	10/72
	Bacteremia (number/total)	13/81	12/72
	Infected ascites (number/total)	1/81	2/72
	Pneumonia (number/total)	7/81	8/72
	New onset organ failure (number/total)	12/81	12/72
	Bowel ischemia (number/total)	2/81	1/72
	ICU admission (number/total)	21/81	15/72

Qualitative summary

A qualitative summary of included papers is presented in Table 1.

Quantitative analysis

Total four studies meeting criteria were selected for quantitative synthesis.

Mortality

**Figure 3:**
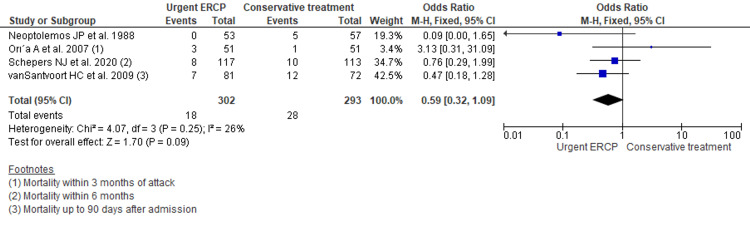
Forest plot comparing mortality outcome across urgent ERCP and conventional approach for acute biliary pancreatitis without cholangitis ERCP: endoscopic retrograde cholangiopancreatography; M-H: Mantel-Haenszel; CI: confidence interval; df: degrees of freedom Four studies reported the mortality outcomes [7,9-11].

There was no significant difference between the two groups when comparing the mortality (in 3-6 months) of urgent ERCP with a conventional approach for acute biliary pancreatitis without cholangitis. However, there was slight lesser mortality among the ERCP group (OR 0.59, 95% CI 0.32 to 1.09; p=0.09; n= 595; I^2^ = 26%) (Figure 3).

Sensitivity analysis was carried out by excluding a non-randomized controlled trial (vanSantvoort HC et al.), a study carried before 2000, and using a random-effect model showed no significant changes in the result (Appendix 3-5).

Overall major complications

**Figure 4:**
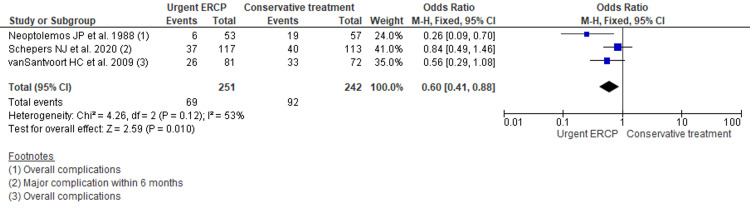
Forest plot comparing the occurrence of complications across urgent ERCP and conventional approach for acute biliary pancreatitis without cholangitis ERCP: Endoscopic retrograde cholangiopancreatography; M-H: Mantel-Haenszel; CI: Confidence interval; df: degrees of freedom Three studies reported the complications [7,10,11].

Three papers reported overall complications in their study. Pancreatic necrosis, new-onset persistent organ failure, bacteremia, cholangitis, pneumonia, or pancreatic insufficiency were considered as major complications. Pooling the data using fixed-effect model showed reduced major complications among urgent ERCP group comparing with conventional approach for acute biliary pancreatitis without cholangitis (OR 0.60, 95% CI 0.41 to 0.88; p=0.010; n= 493; I2 = 53%) (Figure 4). Considering moderate heterogeneity and re-running the analysis using random-effect model could not reach level of significance (OR 0.56, 95% CI 0.30 to 1.01; p=0.05; I2 = 53%) (Appendix 6). Similarly, performing sensitivity analysis by excluding studies before 2000 and excluding non-randomized controlled trials also did not reach statistical significance across the two groups (Appendix 7, 8).

New-onset organ failure

**Figure 5:**
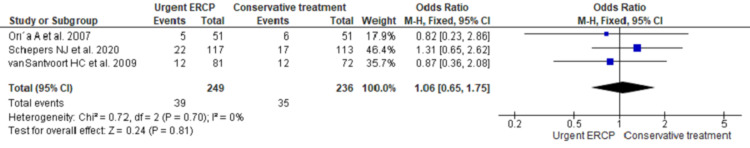
Forest plot comparing the occurrence of new-onset organ failure across urgent ERCP and conventional approach for acute biliary pancreatitis without cholangitis ERCP: endoscopic retrograde cholangiopancreatography; M-H: Mantel-Haenszel; CI: confidence interval; df: degrees of freedom Three studies reported new-onset organ failure [9-11].

Pooling the data using the fixed-effect model for new-onset organ failure among urgent ERCP group compared with a conventional approach for acute biliary pancreatitis without cholangitis showed no significant differences across two groups (OR 1.06, 95% CI 0.65 to 1.75; p=0.81; I2 = 0%) (Figure 5). In addition, subgroup analysis taking specific organ failure and sensitivity analysis carried out by excluding vanSantvoort HC et al. showed no significant changes (Appendix 9, 10).

Pancreatic necrosis

**Figure 6:**
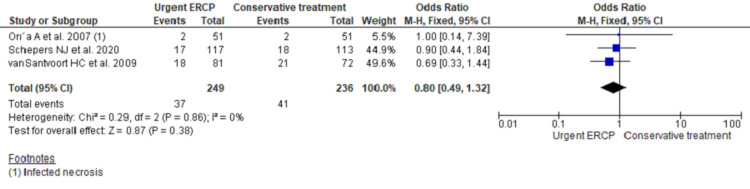
Forest plot comparing the occurrence of pancreatic necrosis across urgent ERCP and conventional approach for acute biliary pancreatitis without cholangitis ERCP: endoscopic retrograde cholangiopancreatography; M-H: Mantel-Haenszel; CI: confidence interval; df: degrees of freedom Three studies reported pancreatic necrosis [9-11].

Pooling the data using the fixed-effect model for pancreatic necrosis among urgent ERCP group compared with the conventional approach for acute biliary pancreatitis without cholangitis showed no significant differences across the two groups (OR 0.80, 95% CI 0.49 to 1.32; p=0.38; I2 = 0%) (Figure 6). In addition, a sensitivity analysis excluding vanSantvoort HC et al. also showed no significant changes (Appendix 11).

Pancreatic pseudo-cyst

Pooling the data using the fixed-effect model for pancreatic pseudo-cyst among urgent ERCP group compared with the conventional approach for acute biliary pancreatitis without cholangitis showed no significant differences across two groups (OR 0.44, 95% CI 0.16 to 1.24; p=0.12; I2 = 0%) (Appendix 12).

ICU admission

Pooling the data using the fixed-effect model for ICU admission rate among urgent ERCP group compared with the conventional approach for acute biliary pancreatitis without cholangitis showed a slightly higher chance of admission in the ERCP group but did not reach statistical significance (OR 1.64, 95% CI 0.97 to 2.77; p=0.06; I2 = 0%) (Appendix 13).

Pneumonia development

Pooling the data using the fixed-effect model for having pneumonia among the urgent ERCP group compared with the conventional approach for acute biliary pancreatitis without cholangitis showed no significant differences across the groups (OR 0.81, 95% CI 0.40 to 1.65; p=0.56; I2 = 0%) (Appendix 14).

Discussion

The study's significant findings were no differences in mortality, ICU admission, complications like pancreatic necrosis, pseudocyst, pneumonia development, and new-onset organ failure among patients with biliary pancreatitis without cholangitis with early ERCP compared to the control group. Although early ERCP was beneficial in reducing major complications while running the fixed-effect model, the same result was not replicated in the random effect model. The role of endoscopic retrograde cholangiopancreatography (ERCP) in the management of acute biliary pancreatitis with cholangitis is well established as per the European and American society of gastroenterology guidelines [19,20]. However, the current recommendation is to avoid ERCP in the absence of cholangitis and ongoing biliary obstruction as per both societies [19,20]. Although prior meta-analyses were conducted to evaluate the role of ERCP in acute biliary pancreatitis without cholangitis, most of the trials included in the analysis had a small sample size, a small number of patients with severe pancreatitis, delay in initiation of ERCP, non-gallstone etiologies, the inclusion of trials with cases of cholangitis and no proper data separating the outcome of those with and without cholangitis [7,8,13]. Thus, we conducted a meta-analysis including the results of Schepers et al.’s randomized controlled trial, the largest ERCP trial, including patients with severe gallstone pancreatitis. In Schepers et al.'s study, ERCP was done earlier than previous trials, and sphincterotomy was done universally in all patients [10].

We found no difference in mortality among the two groups receiving conservative management and endoscopic retrograde cholangiopancreatography for management of acute biliary pancreatitis without cholangitis. This finding was similar to Petrov et al.'s and Moretti et al.’s finding of no difference in mortality in patients with acute biliary pancreatitis without cholangitis [13,21]. Also, we found a reduction in major complications in patients with biliary pancreatitis without cholangitis undergoing ERCP compared to those receiving conservative management using the fixed-effect model. However, the result showed no significance with the random effect model considering the heterogeneity. Moretti et al. and Van Santvoot HR et al. found a decreased risk of pancreatitis-related complications for patients with predicted severe pancreatitis and severe acute biliary pancreatitis with cholestasis, respectively. However, Petrov et al. found no difference in complications among patients who underwent ERCP compared to conservative management [11,13,21]. Moretti et al. reported no difference in complications in mild acute biliary pancreatitis cases without cholangitis in the two groups [21]. Scheper et al. found no increased risk of respiratory complications with ERCP, as seen in previous trials [10].

Similarly, we found no difference in pneumonia among patients receiving conservative management and patients who underwent ERCP. One of the concerns with early ERCP for managing acute biliary pancreatitis without cholangitis is that ERCP has various complications and our findings of somehow decreased major complications are significant. However, we found no difference in local complications of pancreatitis like pancreatic pseudocyst and necrosis among patients receiving conservative treatment and early ERCP. Another interesting finding seen in Schepers’s and Folsch’s trials is the increased risk of cholangitis in patients undergoing conventional therapy than those undergoing early ERCP [8,10].

A comprehensive literature search was performed with a qualitative assessment of the included studies in our meta-analysis. Our meta-analysis explored the role of early ERCP in biliary pancreatitis without cholangitis, a condition in which an effective treatment modality is still evasive. The latest and largest randomized controlled trial results by Schepers et al. were included in our updated analysis [10]. The findings of our study have important implications for clinical practice because no beneficial role of early ERCP was properly established in acute biliary pancreatitis without cholangitis. However, our study has several limitations. Most of the trials included a low number of patients with severe pancreatitis. In addition, the timing to ERCP was variable among the various trials, variable definition of cholangitis in different included trials, and inclusion of various types of patients with varying severity of pancreatitis, and the presence or absence of cholestasis lead to significant biological heterogeneity. In addition, it is hard to ascertain concomitant cholangitis only based on the Charcot triad because gall stone pancreatitis can also cause fever, and cholangitis may sometimes develop in the absence of fever and jaundice [11]. So, some trials might have included patients with concomitant cholangitis.

## Conclusions

Based on our meta-analysis taking patients with acute biliary pancreatitis without cholangitis, there is no benefit of early ERCP. Early ERCP in acute biliary pancreatitis without cholangitis did not reduce mortality, complications, and other adverse outcomes compared to the conservative treatment.

## Figures and Tables

**Figure 7:**
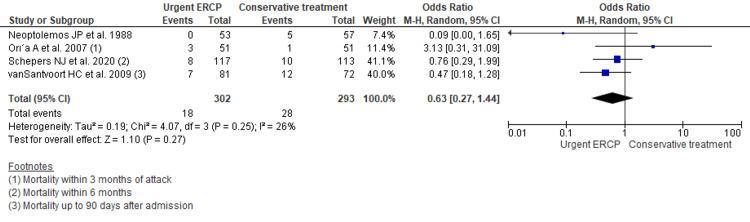
Forest plot comparing mortality outcome across urgent ERCP and conventional approach for acute biliary pancreatitis without cholangitis using a random-effect model ERCP: endoscopic retrograde cholangiopancreatography; M-H: Mantel-Haenszel; CI: confidence interval; df: degrees of freedom Four studies reported the mortality outcomes [7,9-11].

**Figure 8:**
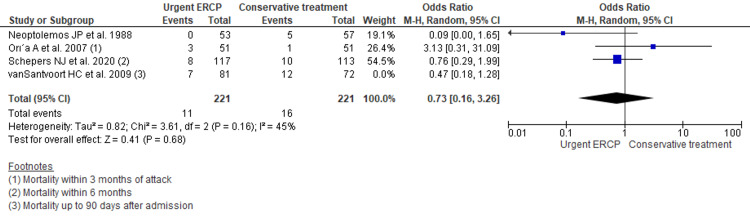
Forest plot comparing mortality outcome across urgent ERCP and conventional approach for acute biliary pancreatitis without cholangitis excluding non-randomized controlled trial (vanSantvoort HC et al.) ERCP: endoscopic retrograde cholangiopancreatography; M-H: Mantel-Haenszel; CI: confidence interval; df: degrees of freedom Three studies included in the forest plot are [7,9,10].

**Figure 9:**
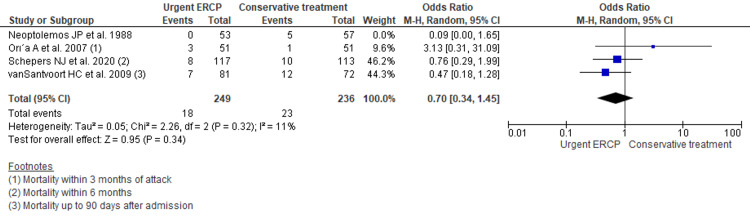
Forest plot comparing mortality outcome across urgent ERCP and conventional approach for acute biliary pancreatitis without cholangitis excluding studies before 2000 (Neoptolemos JP et al. 1988) ERCP: endoscopic retrograde cholangiopancreatography; M-H: Mantel-Haenszel; CI: confidence interval; df: degrees of freedom Three studies included in the Forest plot are [9-11].

**Figure 10:**
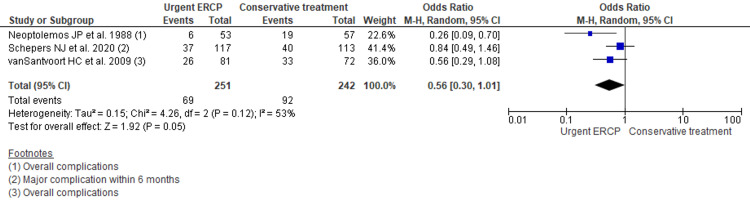
Forest plot comparing the occurrence of complications across urgent ERCP and conventional approach for acute biliary pancreatitis without cholangitis using a random-effect model ERCP: endoscopic retrograde cholangiopancreatography; M-H: Mantel-Haenszel; CI: confidence interval; df: degrees of freedom Three studies reported the complications [7,10,11].

**Figure 11:**
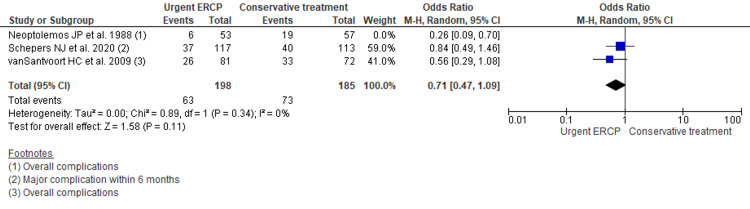
Forest plot comparing the occurrence of complications across urgent ERCP and conventional approach for acute biliary pancreatitis without cholangitis excluding studies before 2000 (Neoptolemos JP et al. 1988) ERCP: endoscopic retrograde cholangiopancreatography; M-H: Mantel-Haenszel; CI: confidence interval; df: degrees of freedom Three studies reported the complications [7,10,11].

**Figure 12:**
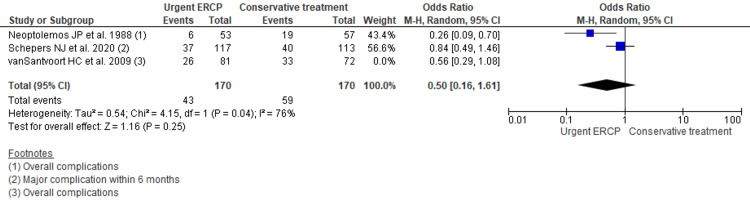
Forest plot comparing the occurrence of complications across urgent ERCP and conventional approach for acute biliary pancreatitis without cholangitis excluding non-randomized controlled trial (vanSantvoort HC et al.) ERCP: endoscopic retrograde cholangiopancreatography; M-H: Mantel-Haenszel; CI: confidence interval; df: degrees of freedom Three studies reported the complications [7,10,11].

**Figure 13:**
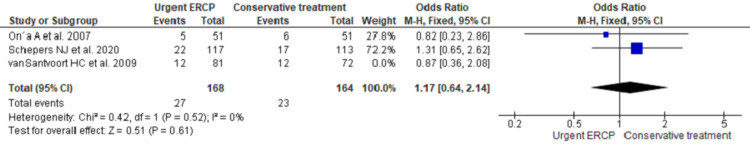
Forest plot comparing the occurrence of new-onset organ failure across urgent ERCP and conventional approach for acute biliary pancreatitis without cholangitis excluding non-randomized controlled trial (vanSantvoort HC et al.) ERCP: endoscopic retrograde cholangiopancreatography; M-H: Mantel-Haenszel; CI: confidence interval; df: degrees of freedom Three studies reported the new-onset organ failure [9-11].

**Figure 14:**
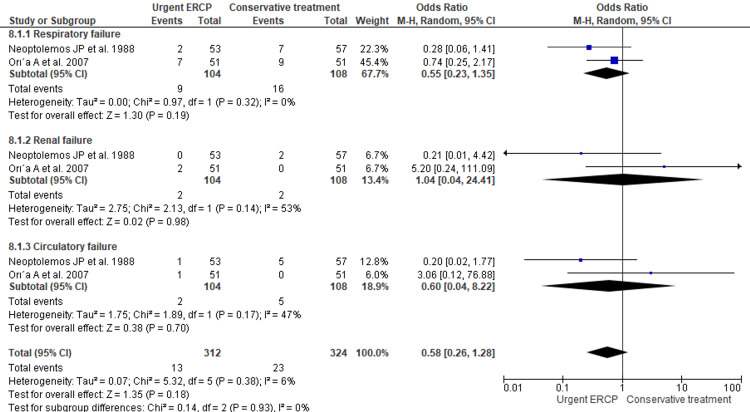
Forest plot showing subgroup analysis on occurrence of specific organ failure across urgent ERCP and conventional approach for acute biliary pancreatitis without cholangitis ERCP: endoscopic retrograde cholangiopancreatography; M-H: Mantel-Haenszel; CI: confidence interval; df: degrees of freedom Two studies reported specific organ failure [7,9].

**Figure 15:**
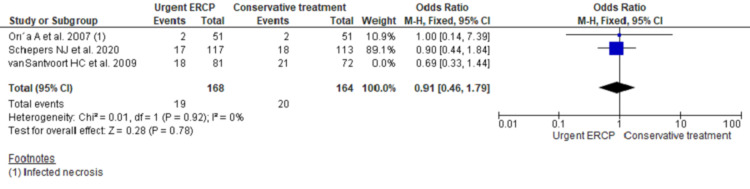
Forest plot comparing the occurrence of pancreatic necrosis across urgent ERCP and conventional approach for acute biliary pancreatitis without cholangitis excluding non-randomized controlled trial (vanSantvoort HC et al.) ERCP: endoscopic retrograde cholangiopancreatography; M-H: Mantel-Haenszel; CI: confidence interval; df: degrees of freedom Three studies reported the pancreatic necrosis [9-11].

**Figure 16:**
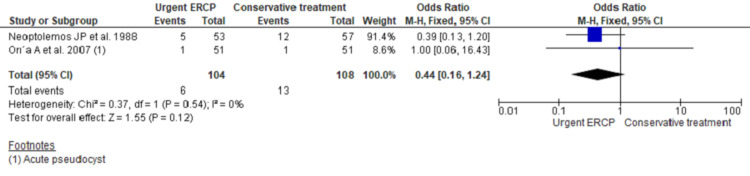
Forest plot comparing the occurrence of pancreatic pseudo-cyst across urgent ERCP and conventional approach for acute biliary pancreatitis without cholangitis ERCP: endoscopic retrograde cholangiopancreatography; M-H: Mantel-Haenszel; CI: confidence interval; df: degrees of freedom Two studies reported pancreatic pseudo-cyst [7,9].

**Figure 17:**
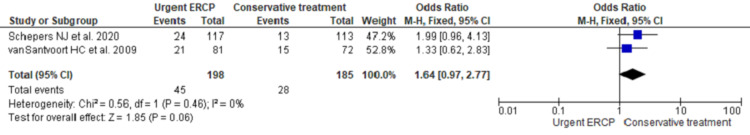
Forest plot comparing ICU admission rate across urgent ERCP and conventional approach for acute biliary pancreatitis without cholangitis ERCP: endoscopic retrograde cholangiopancreatography; M-H: Mantel-Haenszel; CI: confidence interval; df: degrees of freedom Two studies reported ICU admission [10,11].

**Figure 18:**
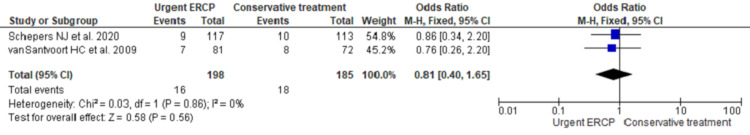
Forest plot comparing the development of pneumonia across urgent ERCP and conventional approach for acute biliary pancreatitis without cholangitis ERCP: endoscopic retrograde cholangiopancreatography; M-H: Mantel-Haenszel; CI: confidence interval; df: degrees of freedom Two studies reported pneumonia [10,11].

## Appendices

Appendix 1

Electronic Search Details: Embase

Search: ('urgent ercp' OR (urgent AND ('ercp'/exp OR ercp)) OR 'endoscopic retrograde cholangio-pancreatography' OR (endoscopic AND retrograde AND 'cholangio pancreatography')) AND ('acute biliary pancreatitis without cholangitis' OR (acute AND biliary AND ('pancreatitis'/exp OR pancreatitis) AND without AND ('cholangitis'/exp OR cholangitis)))

Link: https://www.embase.com/#advancedSearch/resultspage/history.7/page.1/25.items/orderby.date/source.

Total hits: 34

*Electronic Search Details:* *PubMed*

Search: (Urgent ERCP or (Endoscopic retrograde cholangio-pancreatography)) AND (Acute biliary pancreatitis without cholangitis)

Link: https://pubmed.ncbi.nlm.nih.gov/?term=%28Urgent+ERCP+or+%28Endoscopic+retrograde+cholangio-pancreatography%29%29+AND+%28Acute+biliary+pancreatitis+without+cholangitis%29&sort=date

Total hits: 322

*Electronic Search Details:* *PubMed Central*

Search: (Urgent ERCP or (Endoscopic retrograde cholangio-pancreatography)) AND (Acute biliary pancreatitis without cholangitis)

Link: https://www.ncbi.nlm.nih.gov/pmc/?term=(Urgent+ERCP+or+(Endoscopic+retrograde+cholangio-pancreatography))+AND+(Acute+biliary+pancreatitis+without+cholangitis)

Total hits: 2296

*Electronic Search Details:* *Cochrane Library*

Search: Urgent ERCP or Endoscopic Retrograde cholangio-pancreatography in All Text AND Acute biliary pancreatitis without cholangitis or Acute biliary pancreatitis in All Text

Link: https://www.cochranelibrary.com/advanced-search

Total hits: 14

*Electronic Search Details:* *Scopus*

Search: (Urgent ERCP or (Endoscopic retrograde cholangio-pancreatography)) AND (Acute biliary pancreatitis without cholangitis)

Link: https://www.scopus.com/results/results.uri?numberOfFields=0&src=s&clickedLink=&edit=&editSaveSearch=&origin=searchbasic&authorTab=&affiliationTab=&advancedTab=&scint=1&menu=search&tablin=&searchterm1=%28Urgent+ERCP+or+%28Endoscopic+retrograde+cholangio-pancreatography%29%29+AND+%28Acute+biliary+pancreatitis+without+cholangitis%29&field1=TITLE_ABS_KEY&dateType=Publication_Date_Type&yearFrom=Before+1960&yearTo=Present&loadDate=7&documenttype=All&resetFormLink=&st1=%28Urgent+ERCP+or+%28Endoscopic+retrograde+cholangio-pancreatography%29%29+AND+%28Acute+biliary+pancreatitis+without+cholangitis%29&st2=&sot=b&sdt=b&sl=134&s=TITLE-ABS-KEY%28%28Urgent+ERCP+or+%28Endoscopic+retrograde+cholangio-pancreatography%29%29+AND+%28Acute+biliary+pancreatitis+without+cholangitis%29%29&sid=98b53e3b530215da51c640cd717903d4&searchId=98b53e3b530215da51c640cd717903d4&txGid=c6cb180e933f7eaea68f5605edc8353d&sort=plf-f&originationType=b&rr=

Appendix 2

Basic Study Details

The basic details of included studies is presented in Table 2.

**Table 2:** Basic details of included studies APACHE: acute physiology and chronic health evaluation; ERCP: endoscopic retrograde cholangiopancreatography; INR: international normalized ratio; FFP: fresh frozen plasma; PROPATRIA: probiotics in pancreatitis trial; CT: computed tomography; CBD: common bile duct

Study ID	Inclusion criteria	Exclusion criteria
Schepers NJ et al. [10]	Acute pancreatitis	Cholangitis
	High risk of developing severe disease (APACHE II score ≥ 8 OR Modified Glasgow score ≥ 3 OR C-reactive protein > 150 mg/L	Pancreatitis due to other causes such as alcohol abuse (more than four units per day), metabolic causes (hypertriglyceridemia or hypercalcemia), medication, trauma, etc.
	High probability of a biliary etiology	Previous pancreatic sphincterotomy or needle knife pre cut
	Ability to perform ERCP within 24 hours after presentation to the emergency department and no more than 72 hours after symptom onset	Chronic pancreatitis
	In case of a previous episode of necrotizing pancreatitis, patient should be fully recovered	INR that cannot be corrected to less than 1.5 with clotting factors or FFP
	Age ≥18 years	Pregnancy
	Written informed consent	
Neoptolemos JP et al. [7]	Patients admitted with a diagnosis of acute pancreatitis	Pregnancy
		Age < 18 years
		History of chronic alcoholism or acute alcohol intake
		Identifiable secondary cause for the attack of acute pancreatitis, such as drugs, hyperlipidemia, trauma, or surgery
Orı´a A et al. [9]	Patients with a distal main bile duct diameter measuring>=8 mm on admission US	Serious comorbid conditions that precluded ERCP
	Patients with total serum bilirubin>=1.20 mg/dL	Age <18 years
		Pregnancy
		Acute cholangitis
		Inability to perform endoscopy within 72 hours after onset of the attack
vanSantvoort HC et al. [11]	All patients from PROPATRIA diagnosed with acute biliary pancreatitis within 72 hours after onset of symptoms	Other causes of acute pancreatitis (e.g., alcohol abuse)
		Signs of chronic pancreatitis (history and CT)
		Patients with potential cholangitis (serum bilirubin level>1.2 mg/dL and/or dilated CBD on ultrasound or CT and temperature>38.5°C)

An additional analysis was carried out on the following parameters:

1.   Mortality (Appendices 3-5)

2.  Overall major complications (Appendices 6-8)

3.  New-onset organ failure (Appendices 9, 10)

4.  Pancreatic necrosis (Appendix 11)

5.  Pancreatic pseudo-cyst (Appendix 12)

6.  ICU admission (Appendix 13)

7.  Pneumonia development (Appendix 14)

Appendix 3

Sensitivity analysis considering mild heterogeneity and re-running the analysis using random-effect model showed no significant difference across two groups (OR 0.63, 95% CI 0.27 to 1.44; I2 = 26%) (Figure 7).

Appendix 4

Similarly, sensitivity analysis carried out by excluding non-randomized controlled trial (vanSantvoort HC et al.) also showed no significant changes (OR 0.73, 95% CI 0.16 to 3.26; n= 442; I2 = 45%) (Figure 8).

Appendix 5

Additionally, re-running analysis by excluding older studies before 2000 (Neoptolemos JP et al.) also could not show significant changes across two groups (OR 0.70, 95% CI 0.34 to 1.45; I2 = 11%) (Figure 9).

Appendix 6

Considering moderate heterogeneity and re-running the analysis using random effect model could not reach level of significance (OR 0.56, 95% CI 0.30 to 1.01; p=0.05; I2 = 53%) (Figure 10).

Appendix 7

Additionally, re-running analysis by excluding older studies before 2000 (Neoptolemos JP et al.) also could not show significant changes across two groups (OR 0.71, 95% CI 0.47 to 1.09; I2 = 0%) (Figure 11).

Appendix 8

Similarly, sensitivity analysis carried out by excluding non-randomized controlled trials (vanSantvoort HC et al.) also showed no significant changes (OR 0.50, 95% CI 0.16 to 1.61; I2 = 76%) (Figure 12).

Appendix 9

Sensitivity analysis for outcome new-onset organ failure carried out by excluding non-randomized controlled trial (vanSantvoort HC et al.) also showed no significant changes (OR 1.17, 95% CI 0.64 to 2.14; I2 = 0%) (Figure 13).

Appendix 10

Carrying analysis using random effect model for specific organ failure could not reach significant differences among two groups for respiratory failure (OR 0.55, 95% CI 0.23 to 1.35; I2 = 0%), renal failure (OR 1.04, 95% CI 0.04 to 24.41; I2 = 53%), and circulatory failure (OR 0.60, 95% CI 0.04 to 8.22; I2 = 47%) (Figure 14).

Appendix 11

Sensitivity analysis for outcome pancreatic necrosis carried out by excluding non-randomized controlled trial (vanSantvoort HC et al.) also showed no significant changes (OR 0.91, 95% CI 0.46 to 1.79; I2 = 0%) (Figure 15).

Appendix 12

Pooling the data using the fixed-effect model for pancreatic pseudo-cyst among urgent ERCP group comparing with the conventional approach for acute biliary pancreatitis without cholangitis showed no significant differences across two groups (OR 0.44, 95% CI 0.16 to 1.24; p=0.12; I2 = 0%) (Figure 16).

Appendix 13

Pooling the data using the fixed-effect model for ICU admission rate among urgent ERCP group comparing with the conventional approach for acute biliary pancreatitis without cholangitis showed a slightly higher chance of admission in the ERCP group but did not reach statistical significance (OR 1.64, 95% CI 0.97 to 2.77; p=0.06; I2 = 0%) (Figure 17).

Appendix 14

Pooling the data using the fixed-effect model for having pneumonia among the urgent ERCP group compared with the conventional approach for acute biliary pancreatitis without cholangitis showed no significant differences across the groups (OR 0.81, 95% CI 0.40 to 1.65; p=0.56; I2 = 0%) (Figure 18).

## References

[1] Xiao AY, Tan MLY, Wu LM, Asrani VM, Windsor JA, Yadav D, Petrov MS (2016). Global incidence and mortality of pancreatic diseases: a systematic review, meta-analysis, and meta-regression of population-based cohort studies. Lanc Gastro Hepa.

[2] Peery AF, Dellon ES, Lund J (2012). Burden of gastrointestinal disease in the United States: 2012 update. Gastroenterology.

[3] Yadav D, Lowenfels AB (2013). The epidemiology of pancreatitis and pancreatic cancer. Gastroenterology.

[4] Acosta JM, Ledesma CL (1974). Gallstone migration as a cause of acute pancreatitis. N Engl J Med.

[5] Mann DV, Hershman MJ, Hittinger R, Glazer G (1994). Multicentre audit of death from acute pancreatitis. Br J Surg.

[6] Banks PA, Bollen TL, Dervenis C (2013). Classification of acute pancreatitis--2012: revision of the Atlanta classification and definitions by international consensus. Gut.

[7] Neoptolemos JP, Carr-Locke DL, London NJ, Bailey IA, James D, Fossard DP (1988). Controlled trial of urgent endoscopic retrograde cholangiopancreatography and endoscopic sphincterotomy versus conservative treatment for acute pancreatitis due to gallstones. Lancet (London, England.

[8] Fölsch UR, Nitsche R, Lüdtke R, Hilgers RA, Creutzfeldt W (1997). Early ERCP and papillotomy compared with conservative treatment for acute biliary pancreatitis. N Engl J Med.

[9] Oría A, Cimmino D, Ocampo C (2007). Early endoscopic intervention versus early conservative management in patients with acute gallstone pancreatitis and biliopancreatic obstruction: a randomized clinical trial. Ann Surg.

[10] Schepers NJ, Hallensleben NDL, Besselink MG (2020). Urgent endoscopic retrograde cholangiopancreatography with sphincterotomy versus conservative treatment in predicted severe acute gallstone pancreatitis (APEC): a multicentre randomised controlled trial. Lancet.

[11] van Santvoort HC, Besselink MG, de Vries AC (2009). Early endoscopic retrograde cholangiopancreatography in predicted severe acute biliary pancreatitis: a prospective multicenter study. Ann Surg.

[12] Fan ST, Lai EC, Mok FP, Lo CM, Zheng SS, Wong J (1993). Early treatment of acute biliary pancreatitis by endoscopic papillotomy. N Engl J Med.

[13] Petrov MS, van Santvoort HC, Besselink MG, van der Heijden GJ, van Erpecum KJ, Gooszen HG (2008). Early endoscopic retrograde cholangiopancreatography versus conservative management in acute biliary pancreatitis without cholangitis: a meta-analysis of randomized trials. Ann Surg.

[14] Vege SS, DiMagno MJ, Forsmark CE, Martel M, Barkun AN (2018). Initial medical treatment of acute pancreatitis: American Gastroenterological Association Institute Technical Review. Gastroenterology.

[15] Liberati A, Altman DG, Tetzlaff J (2009). The PRISMA statement for reporting systematic reviews and meta-analyses of studies that evaluate healthcare interventions: explanation and elaboration. BMJ.

[16] Shrestha D, Budhathoki P, Poudel A, Adhikari A, Aryal BB, Sedhai YR (2021). Urgent ERCP vs conventional approach in acute biliary pancreatitis without cholangitis: a systematic review and meta-analysis. PROSPERO.

[17] Sterne JA, Savović J, Page MJ (2019). RoB 2: a revised tool for assessing risk of bias in randomised trials. BMJ.

[18] (2019). Cochrane Handbook for Systematic Reviews of Interventions. https://books.google.com/books?hl=en&lr=&id=cTqyDwAAQBAJ&oi=fnd&pg=PR3&dq=Cochrane+handbook+for+systematic+reviews+of+interventions&ots=tviIAeuJih&sig=fExG2bKJkc_xNnj2XGm2RnDlAlQ#v=onepage&q=Cochrane%20handbook%20for%20systematic%20reviews%20of%20interventions&f=false.

[19] Tenner S, Baillie J, DeWitt J, Vege SS (2013). American College of Gastroenterology guideline: management of acute pancreatitis. Am J Gastroenterol.

[20] Arvanitakis M, Dumonceau JM, Albert J (2018). Endoscopic management of acute necrotizing pancreatitis: European Society of Gastrointestinal Endoscopy (ESGE) evidence-based multidisciplinary guidelines. Endoscopy.

[21] Moretti A, Papi C, Aratari A, Festa V, Tanga M, Koch M, Capurso L (2008). Is early endoscopic retrograde cholangiopancreatography useful in the management of acute biliary pancreatitis? A meta-analysis of randomized controlled trials. Dig Liver Dis.

